# Supplementary material for: Anticapsular and Antifungal Activity of α-Cyperone
Source: Antibiotics (Basel). 2021 Jan 6;10(1):51. doi: 10.3390/antibiotics10010051 (PMC7825567; doi:10.3390/antibiotics10010051)
Supplement: Supplementary file 1 [file antibiotics-10-00051-s001.pdf]

antibiotics

MDPI

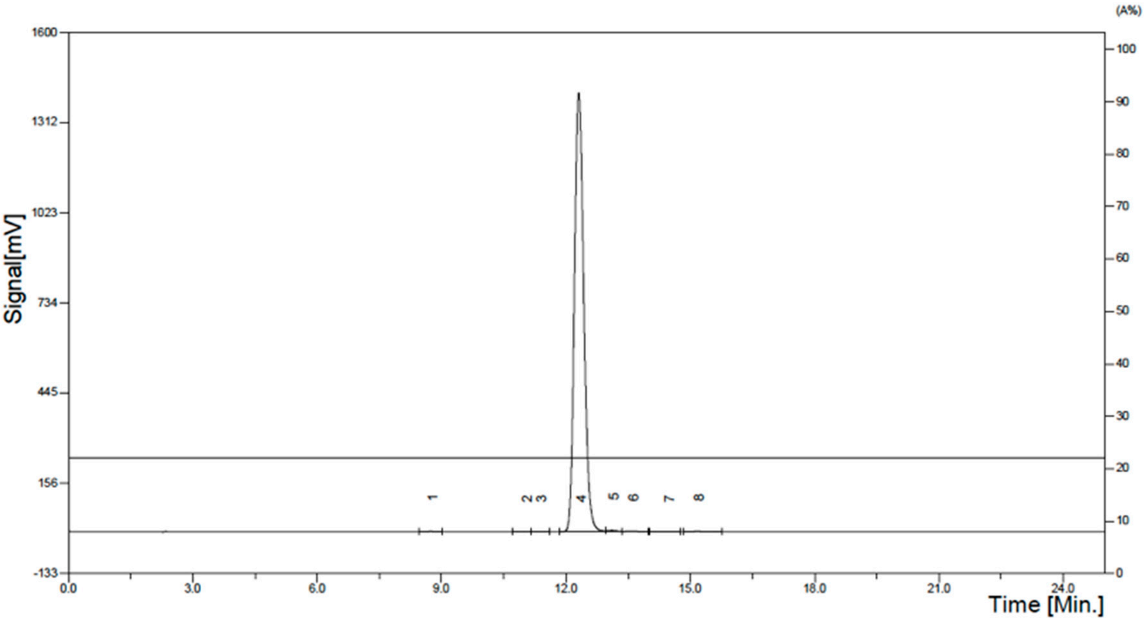

Figure S1. An analytical HPLC chromatogram of α-Cyperone showing a single peak (AdooQ Bioscience, Irvine, CA, USA).

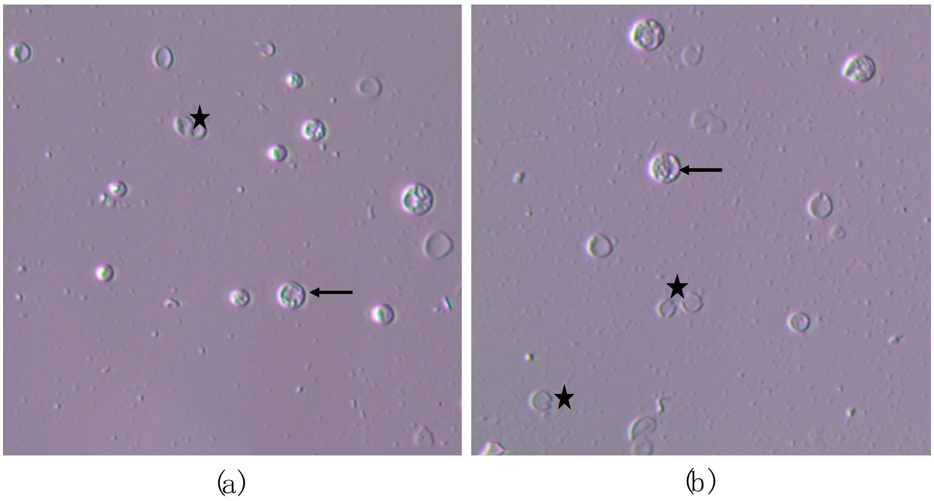

Figure S2. Homogenized capsule-induced cells of control (**a**) and α-Cyperone (16 µg/mL) treated (**b**) *Cryp. neoformans* showing the efficiency of broken cells. Arrow & star symbols indicate unbroken and broken cells, respectively. Homogenizing vials (2 mL) containing cell suspensions + glass beads were shaken in Precellys 24 (Bertin Technologies, France) homogenizer for 15 cycles each for 30 seconds at 5000 rpm speed with a 2-minutes interval between each cycle. Vials were cooled on ice between homogenization cycles. To determine the efficiency of cell breaking, an aliquot of suspensions from each sample was viewed under the microscope (40x obj). Multiple sampling were examined and representative images are shown. While about 50% of cells remain unbroken in the control (*e.g.* 9 out of 18 total in (**a**)), ~80% of the α-Cyperone-treated cells were broken (*e.g.* 11 out of 14 total in (**b**)) which may indicate a weakening of the cell wall/capsule in the treated sample.
